# Azacitidine as maintenance therapy in pediatric *de novo* acute myeloid leukemia

**DOI:** 10.3389/fimmu.2025.1696125

**Published:** 2025-12-05

**Authors:** Chunping Wu, Chunxia Cai, Mei Li, Nainong Li, Yongzhi Zheng, Hao Zheng

**Affiliations:** 1 Department of Paeditric Hematology, Fujian Institute of Hematology, Fujian Provincial Key Laboratory on Hematology, Fujian Medical University Union Hospital, Fuzhou, China; 2 Department of Hematology, Fujian Institute of Hematology, Fujian Provincial Key Laboratory on Hematology, Fujian Medical University Union Hospital, Fuzhou, China

**Keywords:** pediatric AML, azacitidine maintenance, measurable residual disease, molecular relapse, risk stratification

## Abstract

**Background:**

Relapse remains a major challenge in pediatric acute myeloid leukemia (AML), particularly in patients ineligible for hematopoietic stem cell transplantation (HSCT). Hypomethylating agents like azacitidine are hypothesized to target residual disease, but their efficacy and safety as maintenance therapy in *de novo*pediatric AML require validation.

**Methods:**

In this retrospective cohort study, 78 pediatric patients with *de novo*AML in remission after the C-HUANAN-AML 15 protocol were assigned to either azacitidine maintenance (n=27; subcutaneous 75 mg/m²/day, days 1-14 per cycle for 6 cycles) or observation (n=51) groups. Measurable residual disease (MRD) was monitored longitudinally via multiparameter flow cytometry (<0.1% threshold) and PCR for fusion transcripts. Key outcomes included event-free survival (EFS), overall survival (OS), cumulative incidence of relapse (CIR), and safety.

**Results:**

At a median follow-up of 34.6 months, azacitidine maintenance showed comparable EFS (77.7% vs. 77.0%, p = 0.688), OS (89.7% vs. 85.0%, p=0.368) and CIR (22.3% vs. 21.0%, p=0.838) to observation in the overall cohort. Subgroup analysis suggested a non-significant trend toward improved EFS (85.1% vs. 69.3%, p=0.198) and OS (92.9% vs. 81.6%, p=0.304) in intermediate-risk patients. However, among the 17 patients with core-binding factor AML (CBF-AML) and baseline fusion transcripts ≥0.1%, azacitidine maintenance (n=9) showed significantly superior EFS and CIR compared to observation (n=8) (EFS: 100% vs. 62.5%, p=0.048; CIR: 0.0% vs. 40.0%, p = 0.042). Although 40.7% of patients experienced grade 2-4 myelosuppression, all completed the treatment without dose reductions.

**Conclusion:**

Azacitidine maintenance therapy may sustain molecular remission in specific subgroups of pediatric AML, particularly CBF-AML patients with persistent MRD after induction who are ineligible for HSCT. However, the potential benefits must be weighed against the toxicity profile. The optimal dosing and scheduling require further investigation, and broader application warrants validation through larger, prospective, randomized controlled trials.

## 1 Introduction

Survival outcomes for pediatric acute myeloid leukemia (AML) continue to present a significant challenge, with 5-year overall survival (OS) rates plateauing at approximately 70% in recent years despite therapeutic advances (1–3). Relapse remains the primary obstacle to cure, affecting more than 30–40% of children with intermediate (IR)- or high-risk AML, and is frequently associated with poor salvage rates due to both chemoresistance and treatment abandonment (4, 5). While hematopoietic stem cell transplantation (HSCT) may reduce relapse risk in eligible pediatric AML patients, its clinical application is constrained by significant risks, including treatment-related mortality exceeding 20% in some cohorts, life-threatening graft-versus-host disease affecting organ function and quality of life, substantial financial burdens, and potential long-term complications such as infertility and secondary malignancies (6). This underscores the urgent need for alternative relapse prevention strategies for non-transplant candidates and high-risk subgroups.

Maintenance therapy has improved outcomes in pediatric acute lymphoblastic leukemia but has yielded inconsistent results in AML (7, 8). Historical approaches employing chemotherapeutic agents (e.g., AML-BFM protocols, CCLG-AML 2015 protocol) or low-dose cytarabine have failed to demonstrate survival benefits in pediatric AML, potentially because of insufficient targeting of leukemia stem cells and limited immunomodulatory effects (9, 10). Emerging evidence suggests that epigenetic dysregulation and immune evasion play pivotal roles in AML relapse (5, 11). Hypomethylating agents (HMAs), particularly azacitidine, have shown dual antileukemic activity through DNA demethylation-mediated tumor suppression and enhanced antitumor immunity by upregulating tumor-associated antigens and immune checkpoint modulation (12). The QUAZAR AML trial showed that azacitidine maintenance significantly improved long-term survival outcomes in older/unfit patients with AML, with correlative studies demonstrating restored T-cell effector function and natural killer cell activation (13, 14). Based on clinical evidence from QUAZAR, oral azacitidine is recommended by both the European LeukemiaNet (ELN) and the National Comprehensive Cancer Network (NCCN) for the maintenance of patients with AML (15). Notably, in settings where oral azacitidine is unavailable, the NCCN and ELN guidelines recommend injectable azacitidine as a substitute for oral azacitidine (15).

Despite these advances, the immunological effect of HMAs in pediatric AML remains unexplored. Pediatric AML exhibits distinct epigenetic landscapes compared to its adult counterparts, and the developing immune system may respond differentially to HMAs (16). Notably, the safety profile and therapeutic benefits of azacitidine maintenance therapy in pediatric patients with AML remain underexplored. In this study, we investigated subcutaneous azacitidine maintenance therapy in pediatric patients with AML who were ineligible for HSCT. We hypothesized that extended epigenetic immune modulation via azacitidine could improve disease-free survival (DFS) by targeting leukemia stem cell reservoirs.

## 2 Patients and methods

### 2.1 Patients

We enrolled pediatric patients with *de novo* AML treated using the C-HUANAN-AML 15 protocol. The diagnosis of AML was confirmed on the basis of the World Health Organization classifications (17). The cohort focused on non-transplant-eligible populations to investigate the immunomodulatory correlates of epigenetic maintenance therapy. The inclusion criteria were as follows: (i) achievement of complete remission after two fludarabine, cytarabine, granulocyte colony-stimulating factor, and idarubicin (FLAG-IDA) induction cycles and (ii) completion of two high-dose cytarabine-based consolidation cycles. The exclusion criteria were as follows: (i) allo-HSCT in the first complete remission (CR1), (ii) acute promyelocytic leukemia (FAB-M3), (iii) prior exposure to cytotoxic therapy before diagnosis of AML, and (iv) Down syndrome-associated AML due to distinct treatment responses and immune profiles. The institutional review boards of Fujian Medical University Union Hospital approved the study protocol (2025KY288). Written informed consent was obtained from guardians in accordance with the Declaration of Helsinki, and serial immunological monitoring was integrated into the consent framework.

### 2.2 Treatment

The C-HUANAN-AML-15 protocol (18), adapted from the UK MRC AML15 trial (19), is a four-cycle regimen consisting of (i) two sequential FLAG-IDA or DAE (daunorubicin, Ara-C, and etoposide) induction courses, (ii) Homosophocarpine-cytarabine consolidation, and (iii) mitoxantron-cytarabine consolidation. The treatment schematics are shown in **Supplementary Figure S1**. Following the completion of standard chemotherapy, patient assignment to the study groups was non-randomized and conducted as follows. In accordance with the principles of the Declaration of Helsinki, patients and their guardians were fully informed about the potential benefits and risks of azacitidine maintenance therapy. Written informed consent was obtained from those who opted for this intervention. Patients whose families consented received azacitidine maintenance (azacitidine group), while those who declined entered the observation group. A retrospective analysis was then performed on the clinical data of these two cohorts. Maintenance therapy with azacitidine is initiated under the following conditions: within 120 days after completing consolidation therapy, upon hematologic recovery (neutrophil count ≥1.5×10^9^/L and platelet count ≥100×10^9^/L). The azacitidine maintenance group received subcutaneous azacitidine (75 mg/m²/day for 14 days per 28-day cycle, repeated for six cycles), while the observation group underwent routine surveillance without additional therapy.

### 2.3 Risk stratification for survival analysis

Risk stratification was performed based on the criteria outlined in **Supplementary Table S1**, which integrated genomic aberrations and post-induction measurable residual disease (MRD) status. Longitudinal monitoring of MRD was performed using multiparameter flow cytometry in all patients, with parallel quantitative PCR assays targeting leukemia-specific genomic aberrations (e.g., *RUNX1::RUNX1T1*, *CBFB::MYH11*, *FLT3-ITD*, *NPM1* mutations) in patients with fusion genes or actionable mutations. This classification system, aligned with the updated 2022 ELN recommendations (20) and NCCN guidelines (21), was further refined to address pediatric-specific genomic vulnerabilities and dynamic immune reconstitution patterns following intensive chemotherapy (22, 23).

### 2.4 Definitions

MRD negativity thresholds were defined as <0.1% leukemic blasts by multiparameter flow cytometry (sensitivity 10^-4^) and <0.01% by quantitative reverse transcription PCR (sensitivity 10^-4^), in alignment with EuroMRD consortium guidelines (24, 25). Grade 3 or higher adverse events, as defined by the Common Terminology Criteria for Adverse Events version 5.0, were classified as serious adverse events. Treatment-related mortality encompasses deaths directly attributable to serious adverse events of chemotherapy. Event-free survival (EFS) was calculated from diagnosis to the first event or last follow-up. Events included (i) relapse (reappearance of blasts post-remission), (ii) death from any cause, (iii) treatment abandonment (failure to complete curative-intent therapy), and (iv) secondary malignancy. OS was defined as the interval from the initial diagnosis to death or the last known follow-up. The cumulative incidence of relapse (CIR) was defined from the time of CR to relapse, with treatment-related mortality in the absence of relapse considered competing events. Patients were followed up until death, last contact, or censoring at the study cutoff (May 21, 2025).

### 2.5 Statistical analysis

Data were analyzed using SPSS version 28.0 (IBM Corp., Armonk, NY, USA) and GraphPad Prism version 7 (GraphPad Software Inc., San Diego, CA, USA). Descriptive statistics for continuous variables, reported as median (range), were compared using nonparametric Mann–Whitney U tests. Associations between categorical variables were evaluated with either χ² or Fisher’s exact tests based on contingency table suitability. Survival analysis was performed using the Kaplan–Meier method and log-rank comparisons. Prognostic factors were identified using univariate Cox regression (variables with *p*<0.05), followed by multivariate Cox proportional hazards modeling. All statistical tests were bilateral, with a significance threshold set at a *p*-value of less < 0.05.

## 3 Results

### 3.1 Patient characteristics

**Figure 1:**
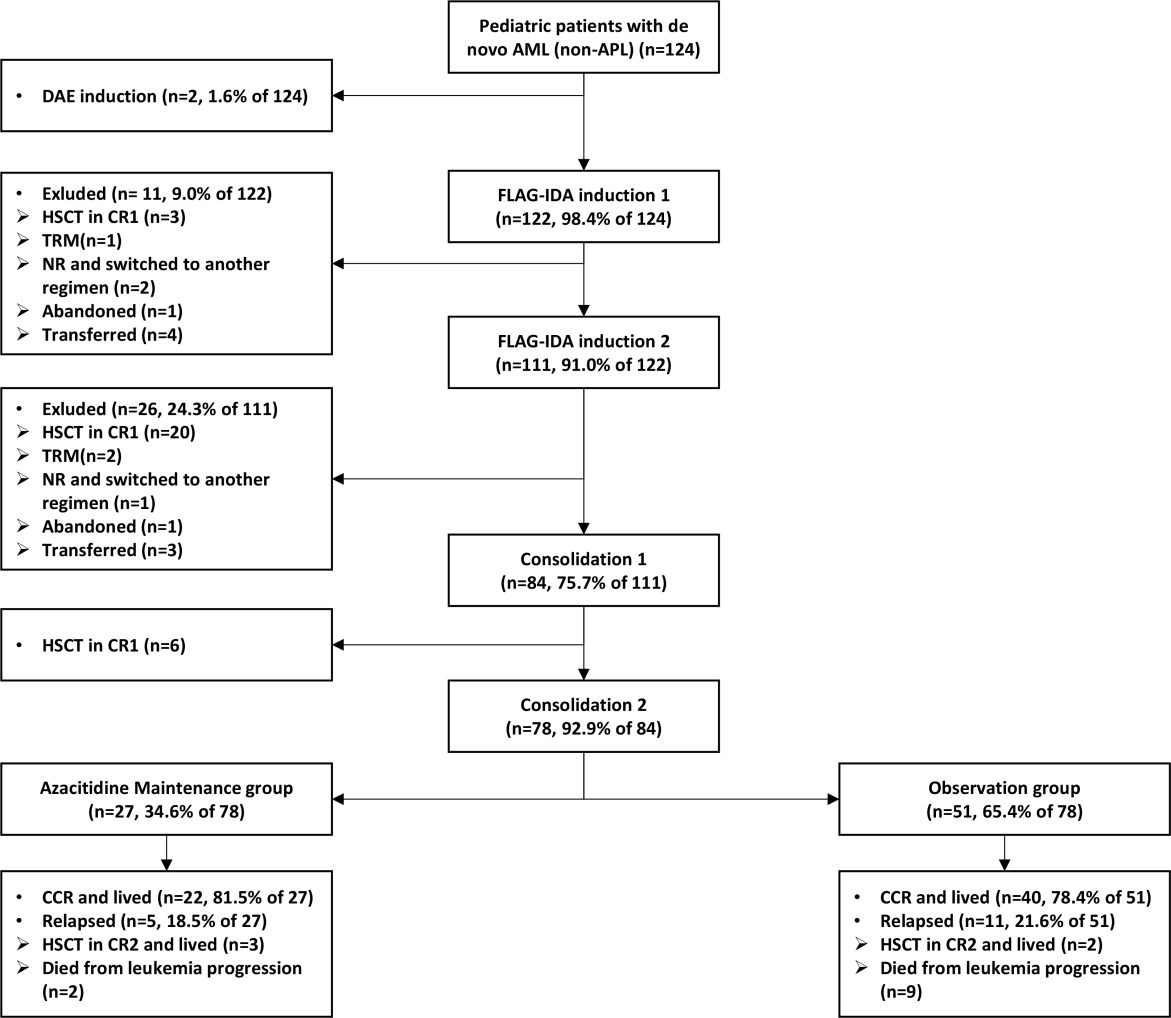
Patient selection flowchart and treatment results. AML, acute myeloid leukemia; FLAG-IDA, fludarabine, cytarabine, granulocyte colony-stimulating factor, and idarubicin; HSCT, hematopoietic stem cell transplantation; TRM, treatment-related mortality; CR, complete remission; CR1, the first complete remission; CR2, the second complete remission; CCR, continuous complete remission.

Between January 2020 and December 2024, 124 pediatric patients (aged <14 years) with *de novo* AML were treated using the C-HUANAN-AML 15 protocol. **Figure 1** outlines the treatment flow. Forty-six patients were excluded from the analytic cohort: 2 received DAE induction, 3 experienced treatment-related mortality, 9 abandoned treatment or transferred care, 3 failed to achieve remission, and 29 underwent HSCT in CR1. The 78 included patients completed four chemotherapy cycles (two FLAG-IDA inductions followed by two high-dose cytarabine consolidations) comprised the final study cohort, which included 13 patients with low risk (LR), 41 with intermediate risk (IR), and 24 with high risk (HR). Among the 24 patients with HR—who constituted the population for whom HSCT was specifically indicated but was declined by their families—the adverse prognostic features were as follows: 14 harbored unfavorable genomic alterations (e.g., monosomy 7, complex karyotype), 7 had *KMT2A*-rearrangements (excluding *MLLT3* partners), 2 carried *FLT3*-ITD mutations, and 2 had *TP53* mutations. Additionally, 10 patients were classified as HR due to a suboptimal response to induction chemotherapy (MRD ≥1% after course 1 or ≥0.1% after course 2). From this final cohort, 27 patients were allocated to azacitidine maintenance therapy and 51 to observational follow-up. Baseline clinical and genomic characteristics and post-induction risk stratification of the azacitidine maintenance group are detailed in **Supplementary Tables S2**, **S3**. Comparative analysis of baseline clinical parameters, genomic aberrations, and final risk stratification between the azacitidine maintenance group and observation group revealed no significant intergroup differences in age, sex, white blood cell counts at diagnosis, prognosis-associated fusions or mutations (e.g., *RUNX1::RUNXT1*, *CBFβ::MYH11*, *KMT2A* rearrangements, *FLT3*-ITD, and *NPM1* mutation), or distribution across risk categories (**Table 1**).

**Table 1:** Comparison of the azacitidine maintenance and observation groups .

Characteristic	Category	Azacitidine maintenance group (n=21)		Observation group (n=51)		χ^>^	*P* value
		No.	%	No.	%		
Age	≥ 10 years	9	33.3	16	31.4	0.031	0.860
	< 10 years	18	66.7	35	68.6		
Gender	Male	9	33.3	28	54.9	3.294	0.070
	Female	18	66.7	23	45.1		
WBC at diagnosis	≥ 50×10^9^/L	9	33.3	18	35.3	0.030	0.863
	< 50×10^9^/L	18	66.7	33	64.7		
FAB classification	AMKL	2	7.4	1	2.0	^a^/	0.274
	Non-AMKL	25	92.6	50	98.0		
*RUNX1::RUNX1T1*	Positive	8	29.6	21	41.2	1.008	0.315
	Negative	19	70.4	30	58.8		
*CBFβ::MYH11*	Positive	2	7.4	6	11.8	^a^/	0.707
	Negative	25	92.6	45	88.2		
*KMT2A* rearrangements	Positive	3	11.1	10	19.6	^a^/	0.525
	Negative	24	88.9	41	80.4		
*C-KIT* mutation	Positive	5	18.5	13	25.5	0.483	0.487
	Negative	22	81.5	38	74.5		
*FLT3-ITD* mutation	Positive	0	0.0	2	3.9	^a^/	0.541
	Negative	27	100.0	49	96.1		
*ASXL1* mutation	Positive	1	3.7	1	2.0	^a^/	1.000
	Negative	26	96.3	50	98.0		
*NPM1* mutation	Positive	2	7.4	0	0.0	^a^/	0.117
	Negative	25	92.6	51	100.0		
Biallelic mutated *CEBPA* or single bZIP domain mutation	Positive	2	7.4	2	3.9	^a^/	0.606
	Negative	25	92.6	49	96.1		
-7/7q^-^	Positive	0	0.0	2	3.9	^a^/	0.541
	Negative	27	100.0	49	96.1		
^b^Complex	Positive	3	11.1	3	5.9	^a^/	0.412
karyotypes	Negative	24	88.9	48	94.1		
Initial risk	LR	3	11.1	10	19.6	1.929	0.381
stratification	IR	17	63.0	24	47.1		
	HR	7	25.9	17	33.3		

^a^Fisher’s exact tests; ^b^a complex karyotype is defined as the presence of ≥3 unrelated clonal chromosomal abnormalities in the absence of recurrent disease-defining translocations or inversions. WBC, white blood cell count; FAB, French-American-British

### 3.2 MRD monitoring and long-term outcomes

**Supplementary Table S4** summarizes MRD dynamics in the azacitidine maintenance group (*n* = 27). All patients maintained multiparameter flow cytometry-MRD negativity (<0.1%) at baseline and throughout the treatment. By quantitative PCR, six patients were positive for fusion transcripts or mutant *NPM1* prior to maintenance therapy: five with *RUNX1::RUNX1T1* (Cases 1, 4, 8, 22, 26), one with *CBFβ::MYH11* (Case 10), and one with *NPM1* (case 21) cases before azacitidine maintenance. Serial assessments demonstrated that all of the patients with positive molecular MRD achieved PCR-MRD negativity after six maintenance cycles. Although Case 26 (*RUNX1::RUNX1T1*) exhibited low-level recurrence (0.02%) at 4-month post-therapy surveillance, the PCR-MRD test turned negative again upon re-examination one month later. Notably, the two patients (Cases 10 and 21) with pre-maintenance MRD levels >0.1% achieved persistent negativity after therapy.

In the observation group, nine patients had positive PCR-MRD after completing chemotherapy: seven with *RUNX1::RUNX1T1*, one with *CBFB::MYH11*, and one with *KMT2A::MLLT4*. One patient with *RUNX1::RUNX1T1* had an MRD level of 0.14% (the others were <0.1%); transplantation was recommended but declined by the family, and this patient subsequently relapsed. Among the remaining eight, two eventually achieved MRD negativity, while six had persistent low-level positivity.

Among the patients receiving azacytidine maintenance, the relapse rate was 18.5% (5 patients). The median duration before relapse onset was 17.4 months, ranging from 6.8 to 26.4 months. One patient was lost to follow-up after relapse, and one opted for palliative care but later died of leukemia. Three patients underwent reinduction chemotherapy: two achieved a second complete remission and subsequent allo-HSCT and remained leukemia-free; one failed to achieve complete remission, underwent salvage transplantation, had transient complete remission, but relapsed again 6 months post-transplant, and is now living with active disease. In the observation group, 11 patients experienced relapse, with a median time to relapse of 9.0 months (range 4.7–23.6 months). Four patients received reinduction chemotherapy: two achieved a second complete remission and subsequent HSCT and remained leukemia-free, and seven died from leukemia progression after relapse.

### 3.3 Survival analysis

**Figure 2:**
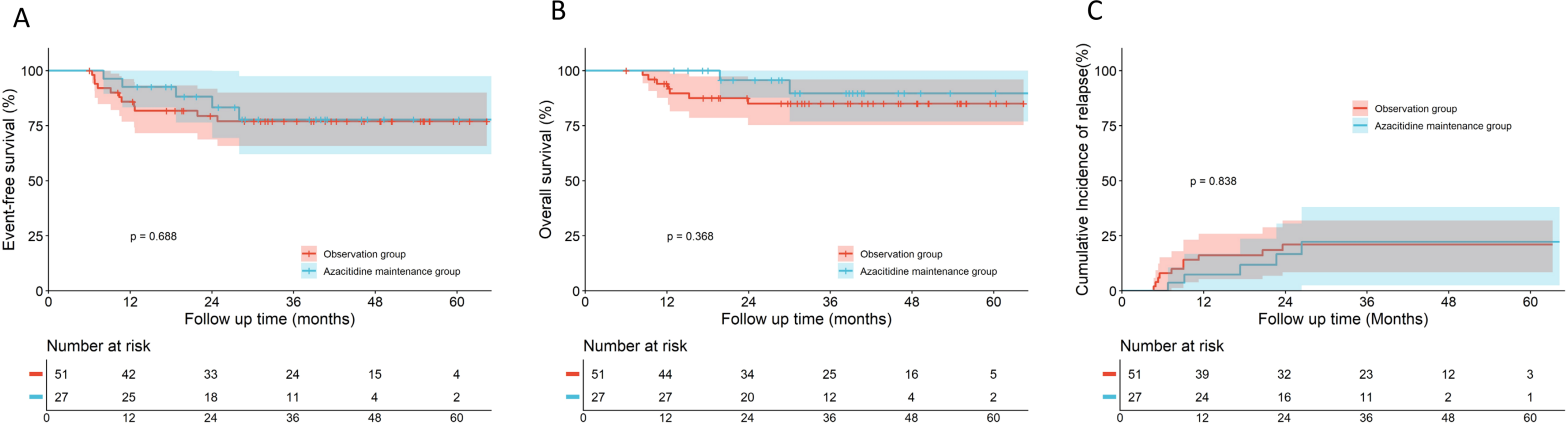
Comparative outcomes between the azacitidine maintenance group and the observation group in the overall cohort. Kaplan–Meier curves for **(A)** Event-free survival, **(B)** Overall survival, and **(C)** Cumulative incidence of relapse.

**Figure 3:**
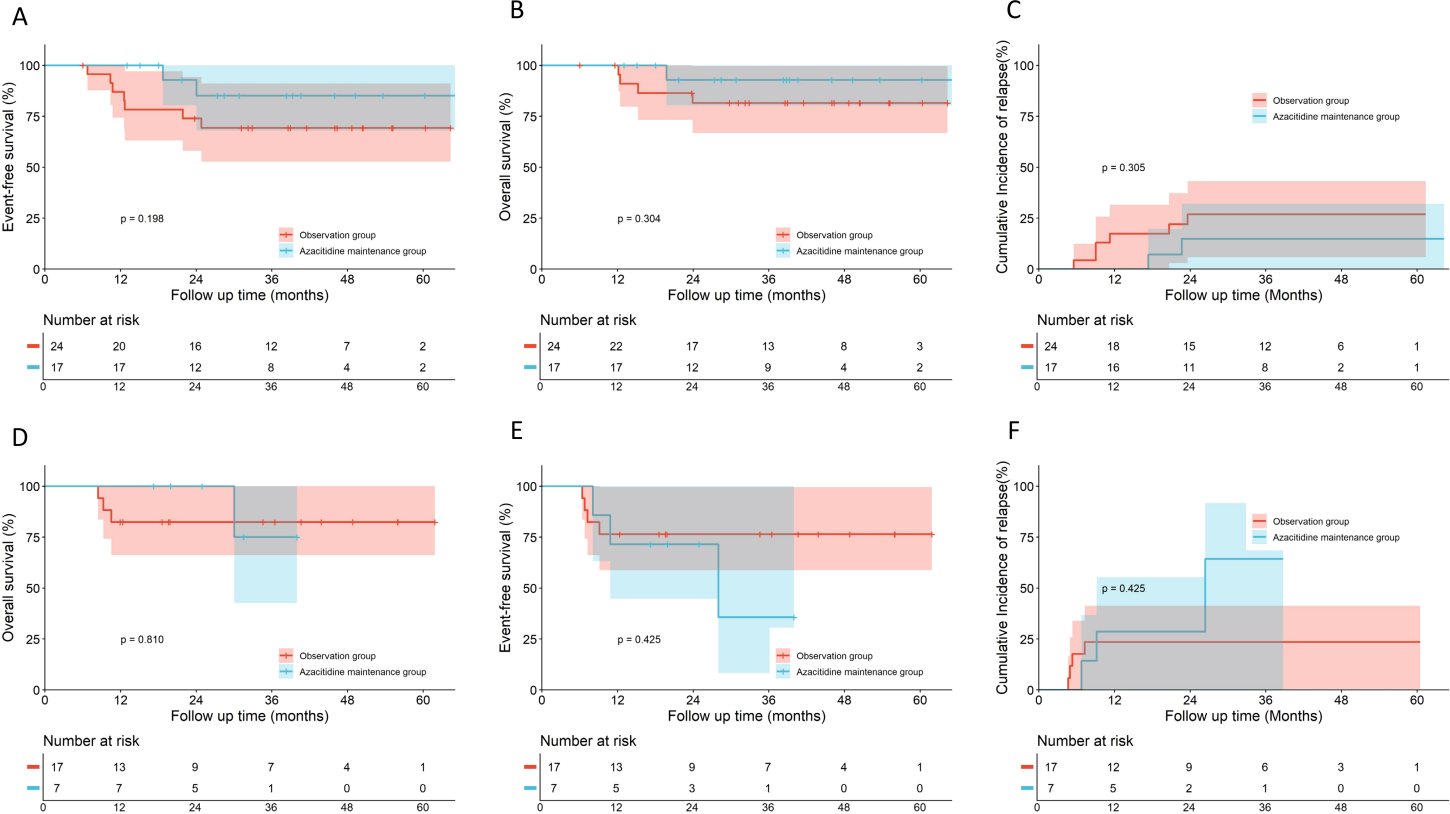
Outcomes of azacitidine maintenance versus observation in pediatric patients with acute myeloid leukemia (AML) stratified by risk. **(A-C)** Kaplan–Meier survival analyses for intermediate-risk (IR) patients (n=41: Azacitidine, n=17; Observation, n=24). **(A)** Event-free survival (EFS); **(B)** Overall survival (OS); **(C)** Cumulative incidence of relapse (CIR); (D-F) Kaplan–Meier survival analyses for high-risk (HR) patients (n=24: Azacitidine, n=7; Observation, n=17). **(D)** EFS; **(E)** OS; **(F)** CIR. Comparisons were made using the log-rank test. The number of patients at risk at each time point is shown below the curves.

**Figure 4:**
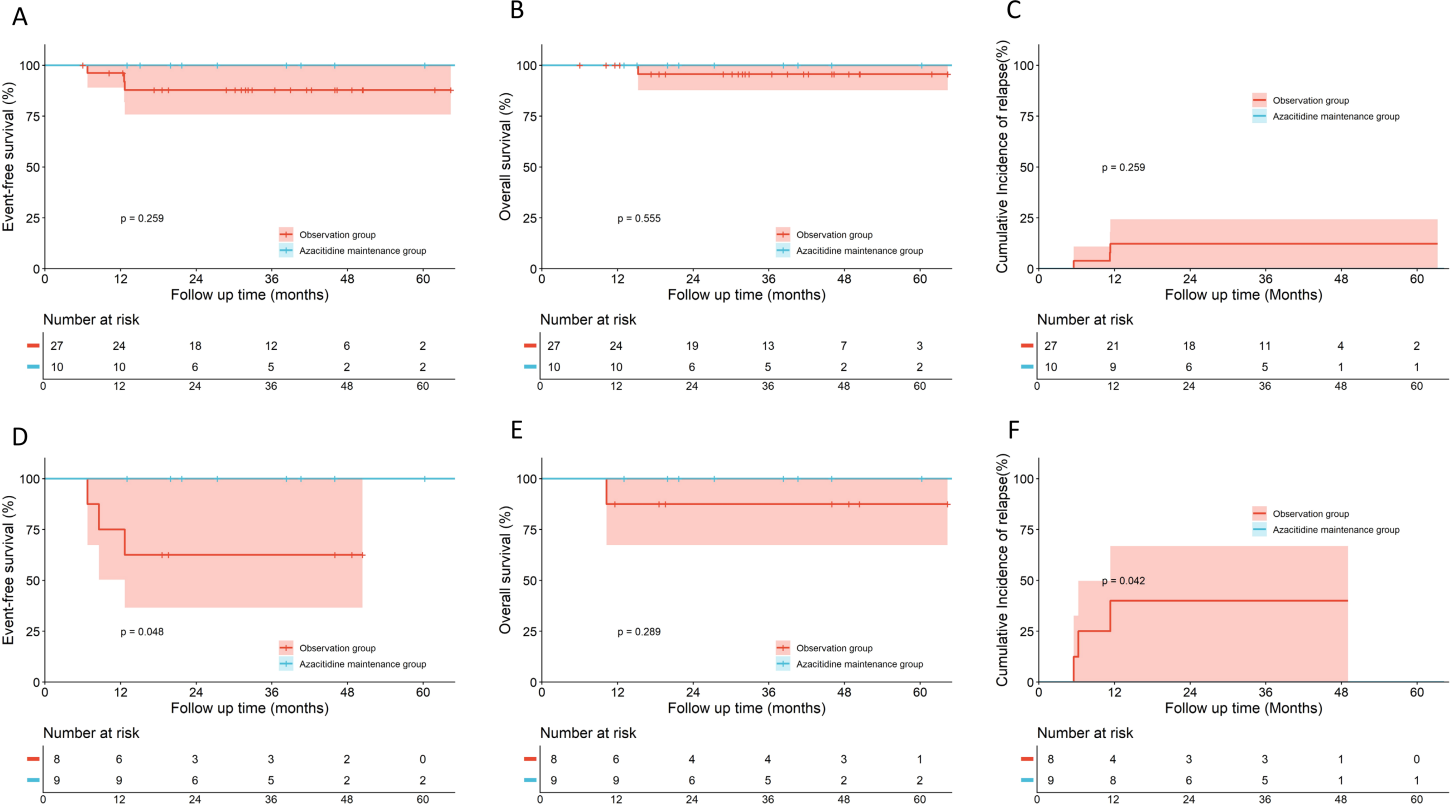
Outcomes of azacitidine maintenance versus observation in pediatric core-binding factor acute myeloid leukemia (CBF-AML). **(A–C)** Analyses for the entire CBF-AML cohort (n=37: Azacitidine, n=10; Observation, n=37). **(A)** Event-free survival (EFS); **(B)** Overall survival (OS); **(C)** Cumulative incidence of relapse **(D–F)** Analyses for the subgroup of patients with CBF-AML with baseline fusion transcript levels ≥0.1% after the first induction cycle (n=17: Azacitidine, n=9; Observation, n=8). **(D)** EFS; **(E)** OS; **(F)** CIR. Comparisons were made using the log-rank test. The number of patients at risk at each time point is shown below the curves.

No significant differences were observed in 5-year EFS, OS, or CIR between the azacitidine maintenance group (n=27) and the observation group in the overall cohort (n=51) (EFS: 77.7% *vs.* 77.0%, hazard ratio [HR], 0.806, 95% confidence interval [CI], 0.290–2.236, *p* = 0.688; OS: 89.7% *vs.* 85.0%, HR, 0.493, 95% CI, 0.127–1.913, *p* = 0.368; CIR: 22.3% *vs.* 21.0%, HR, 0.894, 95% CI, 0.311–2.569, *p* = 0.838) (**Figure 2**). Subgroup analyses by risk stratification (patients with LR were excluded owing to insufficient sample size, compromising statistical power) revealed divergent trends. For the IR subgroup, azacitidine maintenance (n=17) demonstrated a numeric improvement in EFS, OS and CIR compared to observation (n=24), though differences did not reach statistical significance (EFS: 85.1% *vs.* 69.3%, HR, 0.371, 95% CI, 0.099–1.387, *p* = 0.198; OS: 92.9% *vs.* 81.6%, HR, 0.335, 95% CI, 0.057–1.972, *p* = 0.304; CIR:14.9% *vs.* 26.9%, HR, 0.444, 95% CI, 0.110–1.802, *p* = 0.305) (**Figure 3A–C**). For the HR subgroup, no apparent benefit was observed (n=17) with azacitidine maintenance (n=7) (EFS: 35.7% *vs.* 76.5%, HR, 1.817, 95% CI, 0.356–9.265, *p* = 0.425; OS: 75.0% *vs.* 82.4%, HR, 0.758, 95% CI, 0.090–6.362, *p* = 0.810; CIR: 64.3% *vs.* 23.5%, HR, 1.817, 95% CI, 0.356–9.265, *p* = 0.425) (**Figure 3D–F**). Subgroup analysis of core-binding factor AML (CBF-AML) (n=37), the most prevalent molecular subtype, demonstrated no significant differences in EFS, OS, or CIR between groups when analyzing all patients with CBF-AML (EFS: 100% *vs.* 87.8%, HR, 0.240, 95% CI, 0.020–2.859, *p* = 0.259; OS: 100% *vs.* 95.7% ± 4.3%, HR, 0.260, 95% CI, 0.003–22.910, *p* = 0.555; CIR: 0% *vs.* 12.3%, HR, 0.240, 95% CI, 0.020–2.859, *p* = 0.259) (**Figure 4A–C**). However, among 17 patients with baseline fusion transcript levels ≥0.1% after Induction 1, the azacitidine group (n=9) showed significantly superior EFS and CIR compared to observation (n=8) (EFS: 100.0% *vs.* 62.5%, HR, 0.110, 95% CI, 0.010–0.985, *p* = 0.048; CIR: 0% *vs.* 40.0%, HR, 0.091, 95% CI, 0.009–0.913, *p* = 0.042), although OS differences remained non-significant (100% *vs.* 87.5%, HR, 0.119, 95% CI, 0.002–6.061, *p* = 0.289) (**Figure 4D–F**).

### 3.4 Safety of azacitidine

Azacitidine maintenance therapy showed a manageable safety profile in this pediatric cohort. Among 27 patients, 11 (40.7%) experienced myelosuppression: 8 cases of grade 2–3 hematologic toxicities (neutropenia [absolute neutrophil count <1.5 × 10^9^/L], anemia [hemoglobin 7–10 g/dL], thrombocytopenia [platelets 25–50 × 10^9^/L]), and 3 cases of grade 4 neutropenia (absolute neutrophil count <0.5 × 10^9^/L), all managed without red blood cell/platelet transfusions. Non-hematologic adverse events were infrequent and mild: one episode of febrile neutropenia (cycle 1) resolved with piperacillin-tazobactam, and transient grade 1 pruritic rash (n = 1) and self-limiting low-grade fever (<38 °C, n = 1) occurred pre-dose in subsequent cycles, managed with antihistamines and observation, respectively. One case of grade 2 nausea/vomiting was treated with ondansetron. Notably, all patients completed the full 6-cycle regimen with no treatment discontinuation or dose reduction due to toxicity, underscoring the feasibility of prolonged azacitidine administration in children.

## 4 Discussion

Although maintenance therapy has become the standard treatment for pediatric acute lymphoblastic leukemia (26) and acute promyelocytic leukemia (27), its role in the treatment of pediatric AML remains controversial. The AML-BFM regimen series all incorporates maintenance therapy, primarily utilizing chemotherapeutic agents such as mercaptopurine and cytarabine, and serves as a representative model for maintenance therapy in pediatric AML (10, 28). Although the AML-BFM 2012 protocol achieved a 5-year EFS rate of 65% ± 3% and a 5-year OS rate of 82% ± 3%, their reported long-term outcomes do not appear superior to those of protocols without maintenance therapy (2). Similarly, the Chinese CCLG-AML 2015 protocol (9) employed maintenance therapy, yielding 5-year OS and EFS rates of approximately 65% and 60%, respectively; yet, compared to regimens without maintenance therapy (2, 29), it did not demonstrate any significant survival benefit. Owing to uncertainties regarding efficacy, maintenance therapy has not been advocated in most pediatric AML collaborative group protocols for extended periods. 

In recent years, maintenance therapy with non-chemotherapeutic agents, primarily *FLT3* inhibitors and HMAs, has shown survival benefits in adults with AML (7, 12, 14, 30). The rationale for this approach is target-specific: *FLT3*-ITD is one of the most frequent mutations in AML, occurring in approximately 25–30% of adults and 10–15% of pediatric patients, and is associated with high relapse risk and poorer prognosis (30). In adults, post-remission maintenance with *FLT3* inhibitors such as sorafenib (post-HSCT) (31), midostaurin (following induction/consolidation) (32), gilteritinib (post HSCT) (33), and quizartinib (post-HSCT) (34), has demonstrated significant reductions in relapse and improvements in long-term survival. However, experience with *FLT3* inhibitors in pediatric AML remains limited and their benefit is less clear. The Children’s Oncology Group AAML1031 trial, which incorporated sorafenib (200 mg/m²/day) from induction and continued it as single-agent maintenance for up to one year, showed a significant reduction in relapse risk, though without a corresponding OS benefit (35). Moreover, the majority of pediatric AML cases lack actionable targets for currently available inhibitors. Consequently, HMAs, with their established safety profile, tolerability, and considerable clinical experience represent a more universally applicable maintenance option for pediatric AML. This evolving landscape compels a reevaluation of historical conclusions: the lack of survival benefit in earlier maintenance studies may have stemmed from suboptimal drug selection, whereas contemporary strategies incorporating these novel agents hold genuine potential to improve outcomes in pediatric AML. 

This retrospective cohort study provides real-world evidence supporting the feasibility and potential efficacy of azacitidine maintenance therapy in reducing relapse rates in pediatric patients with AML ineligible for HSCT. Although no significant differences in OS or relapse-free survival were observed between the azacitidine and observation groups, our data suggest that risk-adapted MRD-guided maintenance strategies may benefit specific subgroups, particularly those with molecularly persistent CBF-AML.

CBF-AML is characterized by recurrent cytogenetic abnormalities, specifically t (8,21)(q22;q22.1) and inv (16)(p13.1q22)/t (16,16)(p13.1;q22), which generate *RUNX1::RUNX1T1* or *CBFB::MYH11* fusion transcripts, respectively. Although CBF-AML carries a relatively favorable prognosis,suboptimal molecular response after induction therapy—defined by persistent MRD at levels >10^−3^ (or <3-log reduction from baseline)—significantly increases relapse risk in both pediatric and adult patients (36–38). Building upon this risk stratification, recent studies in *adult* patients with CBF-AML have demonstrated that maintenance therapy with HMA (decitabine) significantly improves outcomes in patients with persistent molecular MRD (39, 40). While our study extends the investigation of HMA maintenance to pediatric AML, our findings should be interpreted with caution. In the subset of CBF-AML patients with a suboptimal molecular response (baseline fusion transcripts ≥0.1% after Induction 1), the azacitidine group showed a significantly higher EFS and a lower CIR compared to the observation group. Notably, all six patients with persistent molecular MRD at the end of chemotherapy—two of whom had MRD levels >0.1%—achieved MRD negativity after azacitidine maintenance and remained relapse-free. In stark contrast, the one observation group patient with an MRD level >0.1% experienced an early relapse. These observations may suggest a potential role for azacitidine in eradicating residual disease. However, given the small sample size and the retrospective nature of our study, these results are considered hypothesis-generating, and any conclusion about efficacy remains speculative, warranting validation in larger prospective trials.

Pediatric patients with AML and IR typically face substantial relapse rates of 40–50% and are not routinely considered strong candidates for HSCT in CR1 (1, 3, 22). Although a non-significant trend toward improved EFS and reduce CIR was observed in patients with IR receiving azacitidine maintenance, the lack of a statistically significant benefit in OS, coupled with a grade 2–4 adverse event rate exceeding 40%, necessitates a cautious interpretation. These findings prompt a critical appraisal of the risk–benefit profile of azacitidine maintenance in this specific cohort. Therefore, rather than advocating for its widespread use, our results highlight the need to optimize the dosing and scheduling of azacitidine to improve its tolerability. Future prospective studies should prioritize identifying the patient subgroups most likely to benefit from a better-tolerated maintenance regimen.

Notably, the study showed that azacitidine maintenance therapy may reduce CIR in certain AML subtypes but ultimately failed to confer an OS benefit. This finding aligns with broader patterns observed in adult AML maintenance trials (7). Moreover, in the HR subgroup, outcomes may be worse in patients receiving maintenance therapy. The dissociation between relapse and OS may stem from the mechanisms underlying maintenance therapy. Although maintenance therapy suppresses residual disease to reduce CIR, prolonged treatment, especially without achieving deep molecular responses, may select for resistant subclones by promoting therapeutically driven heterogeneity. Consequently, the efficacy of salvage therapy upon relapse is often significantly compromised, negating potential OS gains despite initial improvements in relapse-free survival.

Key limitations include the retrospective design, single-center patient cohort, and the absence of standardized MRD monitoring across all genetic subtypes. Furthermore, the small high-risk subgroup limited the statistical power to detect differences in survival. Despite these limitations, our findings provide valuable insights to guide future research by identifying specific patient subgroups that may derive the greatest benefits from maintenance therapy.

In conclusion, our study suggests that azacitidine maintenance therapy may confer a benefit in sustaining molecular remission for specific subgroups of pediatric AML, particularly patients with CBF-AML and persistent MRD after induction who are ineligible for HSCT. However, the observed incidence of grade 2–4 myelosuppression underscores that the potential benefits must be weighed against the associated toxicities. The optimal dosing and scheduling of azacitidine in this setting require further investigation. Ultimately, the decision to employ maintenance therapy should be individualized, and its broader application in pediatric AML warrants validation through larger, prospective, randomized controlled trials to definitively establish its risk-benefit profile.

## Data Availability

The raw data supporting the conclusions of this article will be made available by the authors, without undue reservation.
