# Supplementary material for: Azacitidine as maintenance therapy in pediatric de novo acute myeloid leukemia
Source: Front Immunol. 2025 Dec 5;16:1696125. doi: 10.3389/fimmu.2025.1696125 (PMC12714944; doi:10.3389/fimmu.2025.1696125)
Supplement: Supplementary file 1 [file Table1.docx]

Supplementary Material

**Supplementary Table S1.** Risk stratification of pediatric patients with acute myeloid leukemia included in this study

| Risk Category​​ | ​​Cytogenetics/Molecular Genetics​​ | ​​Clinical Characteristics​​ |
| --- | --- | --- |
| ​​Low-risk​​ | 1. t(8;21)(q22;q22.1);*RUNX1::RUNX1T1* 2. inv(16)(p13.1q22);*CBFβ::MYH11* 3. Normal karyotype with *NPM1* mutation 4. Normal karyotype with CEBPA biallelic mutation or single bZIP domain mutation | 1. MFC-MRD <0.1% after first induction 2. Molecular MRD reduction >3-log or transcript level <0.1% after second induction (for *NPM1*/CBF-AML) |
| ​​Intermediate-risk​​ | 1. CBF-AML with c-KIT mutation 2. Cases not meeting low- or high-risk criteria | 1. None specified |
| ​​High-risk​​ | 1. Monosomy 5/7, del(5q/7q) 2. AML transformed from MDS 3. t(3q26.2;v); *MECOM* rearrangement 4. t(v;11q23.3); *KMT2A* rearrangement (excluding t(9;11)) 5. t(6;9)(p23;q34.1); *DEK::NUP214* fusion 6. t(7;12)(q36;p13); *HLXB9::ETV6* fusion 7. t(v;11p15); *NUP98* rearrangements 8. t(9;22)(q34.1;q11.2); *BCR::ABL1* 9. t(16;21)(p11;q22); *TLS::ERG* 10. inv(16)(p13.3;q24.3); *CBFA2T3::GLIS2* 11. t(10;11)(p12;q14); *PICALM::MLLT10* 12. UBTF-TD mutation 13. Complex karyotype ( ≥ 3), monosomal karyotype 14. Mutations in *RUNX1, ASXL1, BCOR, EZH2, SF3B1, SRSF2, STAG2, U2AF1, ZRSR2* 15. *TP53* germline mutation | 1. MFC-MRD ≥1.0% after first induction 2. MFC-MRD ≥0.1% after second induction |

**Supplementary Table S2.** Clinical and genetic characteristics of 27 pediatric patients with de novo AML who received azacitidine maintenance after consolidation

| Case | Sex | Age (month) | WBC at diagnosis (×10^9^/L) | FAB classification | Fusion gene | Chromosome karyotype |
| --- | --- | --- | --- | --- | --- | --- |
| 1 | male | 165 | 4.39 | M2 | *RUNX1::RUNX1T1* | 46, X, -Y, t(8;21)(q22;q22) [20] |
| 2 | male | 32 | 54.85 | M7 | *EVI1* | 47, XY, +mar [1]/46, idem, t(10:11)(p13:q21), -14, add(15)(p11), add(9)(p13) [10]/46,XY [9] |
| 3 | female | 102 | 131.09 | M2 | Negative | 46, XX [20] |
| 4 | male | 57 | 15.58 | M2 | *RUNX1::RUNX1T1* | 46, XY, t(8;21)(q22;q22), del(9)(q22) [9]/46, XY [11] |
| 5 | female | 58 | 9.38 | M2 | Negative | 47, XX, +8 [3]/46, XX [7] |
| 6 | female | 128 | 58.09 | M5 | Negative | 46, XX [20] |
| 7 | female | 136 | 6.47 | M5 | *ZMYND11::MBTD1* | 46, XX [20] |
| 8 | female | 115 | 30.28 | M2 | *RUNX1::RUNX1T1* | 46, X, -X, t(8;21)(q22;q22) [10]/ 46, XX [10] |
| 9 | female | 24 | 6.27 | M2 | *ETV6::TSL* | 46, XX [20] |
| 10 | male | 145 | 102.48 | M5 | *CBFβ::MYH11* | 46, XY, inv(16)(p13q22) [20] |
| 11 | female | 6 | 65.37 | Undetermined | *ETV6::MNX1-AS1* | 48, XX, del(12)(p13), +19 (18)/49, idem, +19[2] |
| 12 | male | 153 | 16.7 | M5 | *MYB::PLEKHO1* | 47, XY, t(1;6)(q21;q23), add(9)(q34), +mar [12]/46, XY [8] |
| 13 | female | 31 | 22.89 | M7 | *NIPBL::HOXB9* | 46,XX,dup(1)(q11q44), del(9)(q13q22), del(12)(p11p13) [7]/46,XX [13] |
| 14 | female | 20 | 5.7 | M5 | *KMT2A::MLLT3* | 46, XX, t(9;11)(p21;q23) [20] |
| 15 | female | 128 | 6.39 | M2 | Negative | 46, XX [20] |
| 16 | female | 87 | 5.29 | M5 | Negative | 46, XX [20] |
| 17 | female | 73 | 54.54 | M5b | Negative | 46, XX [6] |
| 18 | female | 62 | 68.35 | M5a | Negative | 46, XX [20] |
| 19 | female | 146 | 19.7 | M2a | *RUNX1::RUNX1T1* | 46, XX, t(8;21)(q22;q22) [20] |
| 20 | male | 87 | 1.38 | M5b | *KMT2A::MLLT3* | 47, XY, +8 [5]/46,XY [4] |
| 21 | male | 126 | 6.24 | M4 | Negative | 46, XY [18] |
| 22 | female | 157 | 29.88 | M5a | *RUNX1::RUNX1T1* | 42-45, XX, t(8;21)(q22;q22) [CP5]/46,idem, del(9)(q21q32) [8]/46, XX [2] |
| 23 | male | 113 | 293.45 | M5 | *CBFβ::MYH11* | 46, XY [20] |
| 24 | Female | 50 | 62.32 | M5 | *KMT2A::MLLT3* | 46, XX, ?, t(1;9;11)(p36;p21;q23) [20] |
| 25 | male | 111 | 2.96 | M5b | *RUNX1::RUNX1T1* | 45, X, -Y, t(8;21)(q22;q22), del(9)(q31q33) [13]/ 45, -Y, idem [5]/46, XY [3] |
| 26 | Female | 104 | 5.84 | M2 | *RUNX1::RUNX1T1* | 46, XX, t(8;21)(q22;q22) [20] |
| 27 | Female | 96 | 8.61 | M2a | *RUNX1::RUNX1T1* | 46, XX, t(8;21)(q22;q22) [20] |

**Supplementary Table S3.** Measurable residual disease (MRD) assessment, and final risk stratification of 27 pediatric patients with de novo AML who received azacitidine maintenance after consolidation

| Case | Gene mutation | MRD after first induction | | MRD after second induction | | Risk stratification |
| --- | --- | --- | --- | --- | --- | --- |
|  |  | By MFC | By PCR | By MFC | By PCR |  |
| 1 | *KIT* | <0.1% | *RUNX1::RUNXT1* 0.4% | <0.1% | *RUNX1::RUNXT1* 0.1% | IR |
| 2 | *SF3B1, JACK2, MPL, EPPK1* | <0.1% | *EVI1* high expression | <0.1% | *EVI1* low expression | IR |
| 3 | bzip in-frame *CEBPA* mutation | <0.1% | *CEBPA* <0.01% | <0.1% | *CEBPA* <0.01% | LR |
| 4 | Negative | <0.1% | *RUNX1::RUNXT1* 0.19% | <0.1% | *RUNX1::RUNXT1* 0.13% | IR |
| 5 | *NRAS, WT1* | <0.1% | *NRAS* <0.01%*, WT1* low expression | <0.1% | *NRAS* <0.01%*, WT1* low expression | IR |
| 6 | *NPM1, PTPN11, FLT3, IDH2* | <0.1% | *NPM1, PTPN11, FLT3* and *IDH2* <0.01% | <0.1% | *NPM1, PTPN11, FLT3* and *IDH2* <0.01% | LR |
| 7 | *GATA2* | <0.1% | *WT1* high expression | <0.1% | *WT1* high expression | IR |
| 8 | Negative | <0.1% | *RUNX1::RUNXT1* 0.30% | <0.1% | *RUNX1::RUNXT1* 0.12% | IR |
| 9 | Negative | <0.1% | *ETV6::TSL* <0.01% | <0.1% | *ETV6::TSL* <0.01% | IR |
| 10 | *KIT, NRAS, MYC ITD* | <0.1% | *CBFβ::MYH11* 0.42% | <0.1% | *CBFβ::MYH11* 0.31% | IR |
| 11 | *JAK2* | 1% | *WT1* high expression | <0.1% | *WT1* high expression | HR |
| 12 | *NRAS*, *CDKN2A*, *KRAS* | <0.1% | *MYB::PLEKHO1*<0.01% | <0.1% | *MYB::PLEKHO1* <0.01% | IR |
| 13 | *JAK2*, *MPL* | 1.5% | *JAK2* <0.01% | <0.1% | *JAK2* <0.01% | HR |
| 14 | *TP53*, *CBL* | <0.1% | *KMT2A::MLLT3* <0.01%, *TP53* <0.01% | <0.1% | *KMT2A::MLLT3* <0.01%, *TP53* <0.01% | HR |
| 15 | *CEBPA* E59*, *NSD1* | <0.1% | *WT1* high expression, *NSD1* <0.01% | <0.1% | *WT1* low expression;*NSD1* <0.01% | IR |
| 16 | Negative | 1% | *WT1* high expression | <0.1% | *WT1* high expression | HR |
| 17 | monoallelic *CEBPA*, *DNMT3A* | <0.1% | *CEBPA*, *DNMT3A* <0.01% | <0.1% | *CEBPA*, *DNMT3A* <0.01% | IR |
| 18 | Biallelic *CEBPA* | 1.5% | *CEBPA* <0.01% | <0.1% | *CEBPA* <0.01% | HR |
| 19 | *KIT*, *ASXL1*, *FLT3-*TKD | <0.1% | *RUNX1::RUNX1T1* 0.98% | <0.1% | *RUNX1::RUNX1T1* 0.56% | HR |
| 20 | Negative | 1.5% | *KMT2A::MLLT3* <0.01% | <0.1% | *KMT2A::MLLT3* <0.01% | HR |
| 21 | *NPM1*, *PTPN11*, *FLT3*, *GATA2*, *KMT2D* | <0.1% | *NPM1* 0.7% | <0.1% | *NPM1* <0.01% | IR |
| 22 | *ASXL2*, *CBL* | <0.1% | *RUNX1::RUNXT1* 0.28% | <0.1% | *RUNX1::RUNXT1* 0.06% | IR |
| 23 | *FLT3* | <0.1% | *CBFβ::MYH11* 0.37% | <0.1% | *CBFβ::MYH11* 0.03% | IR |
| 24 | *FLT3* | <0.1% | *KMT2A::MLLT3* <0.01% | <0.1% | *KMT2A::MLLT3* <0.01% | IR |
| 25 | *DNM2* | <0.1% | *RUNX1::RUNXT1* 0.10% | <0.1% | *RUNX1::RUNXT1* <0.01% | IR |
| 26 | *KIT* | <0.1% | *RUNX1::RUNXT1* 0.05% | <0.1% | *RUNX1::RUNXT1* <0.01% | IR |
| 27 | *SF3B1, JACK2, MPL, EPPK1* | <0.1% | *RUNX1::RUNXT1* 0.87% | <0.1% | *RUNX1::RUNXT1* 0.05% | IR |

WBC, white blood cell count; FAB, French-American-British

**Supplementary Table S4.** Residual disease (MRD) status was measured using multiparameter flow cytometry (MFC) and quantitative polymerase chain reaction (qPCR) before and after azacitidine maintenance in the maintenance group.

| Case | MRD before azacitidine maintenance | | MRD after azacitidine maintenance | | Long-term outcomes |
| --- | --- | --- | --- | --- | --- |
|  | By MFC | By qPCR | By MFC | By qPCR |  |
| 1 | <0.1% | ***RUNX1::RUNXT1* 0.02%** | <0.1% | ***RUNX1::RUNXT1* <0.01%** | Alive without events |
| 2 | <0.1% | *EVI1* low expression | <0.1% | *EVI1* low expression | Alive without events |
| 3 | <0.1% | *CEBPA* <0.01%, *WT1* high expression | <0.1% | *CEBPA* <0.01%, *WT1* high expression | Alive without events |
| 4 | <0.1% | ***RUNX1::RUNXT1* 0.04%** | <0.1% | ***RUNX1::RUNXT1* <0.01%** | Alive without events |
| 5 | <0.1% | *WT1* low expression | <0.1% | *WT1* low expression | Relapsed, loss |
| 6 | <0.1% | *NPM1* <0.01%; *WT1* low expression | <0.1% | *NPM1* <0.01% | Alive without events |
| 7 | <0.1% | *WT1* high expression | <0.1% | *WT1* high expression | Relapse, alive with relapsed disease (after allo-HSCT) |
| 8 | <0.1% | ***RUNX1::RUNXT1* 0.08%** | <0.1% | ***RUNX1::RUNXT1* <0.01%** | Alive without events |
| 9 | <0.1% | *WT1* high expression | <0.1% | *WT1* high expression | Alive without events |
| 10 | <0.1% | ***CBFβ::MYH11* 0.28%** | <0.1% | ***CBFβ::MYH11* <0.01%** | Alive without events |
| 11 | <0.1% | *WT1* high expression | <0.1% | *WT1* high expression | Relapsed, alive and leukemia-free (after allo-HSCT) |
| 12 | <0.1% | *MYB::PLEKHO1*<0.01% | <0.1% | *MYB::PLEKHO1*<0.01% | Alive without events |
| 13 | <0.1% | *JAK2* <0.01% | <0.1% | *JAK2* <0.01% | Relapsed, alive and leukemia-free (after allo-HSCT) |
| 14 | <0.1% | *KMT2A::MLLT3* <0.01%, *TP53* <0.01% | <0.1% | *KMT2A::MLLT3* <0.01%, *TP53* <0.01% | Alive without events |
| 15 | <0.1% | *WT1* low expression, *NSD1* <0.01% | <0.1% | *WT1* low expression, *NSD1* <0.01% | Alive without events |
| 16 | <0.1% | *WT1* high expression | <0.1% | *WT1* high expression | Relapsed, death due to leukemia progression |
| 17 | <0.1% | *CEBPA*, *DNMT3A* <0.01% | <0.1% | *CEBPA*, *DNMT3A* <0.01% | Alive without events |
| 18 | <0.1% | *WT1* low expression | <0.1% | *WT1* low expression | Alive without events |
| 19 | <0.1% | *RUNX1::RUNXT1* <0.01% | <0.1% | *RUNX1::RUNXT1* <0.01% | Alive without events |
| 20 | <0.1% | *KMT2A::MLLT3* <0.01% | <0.1% | *KMT2A::MLLT3* <0.01% | Alive without events |
| 21 | <0.1% | ***NPM1* 0.7%** | <0.1% | ***NPM1* <0.01%** | Alive without events |
| 22 | <0.1% | ***RUNX1::RUNXT1* 0.02%** | <0.1% | ***RUNX1::RUNXT1* <0.01%** | Alive without events |
| 23 | <0.1% | *CBFβ::MYH11* <0.01% | <0.1% | *CBFβ::MYH11* <0.01% | Alive without events |
| 24 | <0.1% | *KMT2A::MLLT3* <0.01% | <0.1% | *KMT2A::MLLT3* <0.01% | Alive without events |
| 25 | <0.1% | *RUNX1::RUNXT1* <0.01% | <0.1% | *RUNX1::RUNXT1* <0.01% | Alive without events |
| 26 | <0.1% | ***RUNX1::RUNXT1* 0.05%** | <0.1% | ***RUNX1::RUNXT1* <0.01%** | Alive without events |
| 27 | <0.1% | *RUNX1::RUNXT1* <0.01% | <0.1% | *RUNX1::RUNXT1* <0.01% | Alive without events |

Note, Bold indicates PCR-MRD was positive before azacitidine maintenance.

**

**

**Supplementary Figure 1.** Treatment schema for the C-HUANAN-AML15 protocol.
